# Protein-Protein Interaction Among the FoxP Family Members and their Regulation of Two Target Genes, *VLDLR* and *CNTNAP2* in the Zebra Finch Song System

**DOI:** 10.3389/fnmol.2017.00112

**Published:** 2017-05-01

**Authors:** Ezequiel Mendoza, Constance Scharff

**Affiliations:** Institut für Verhaltensbiologie, Freie Universität BerlinBerlin, Germany

**Keywords:** FoxP2, FoxP1, FoxP4, protein interactions, transcription factors, speech, forkhead transcription factors, zebra finch

## Abstract

The Forkhead transcription factor FOXP2 is implicated in speech perception and production. The avian homolog, FoxP2^1^ contributes to song learning and production in birds. In human cell lines, transcriptional activity of FOXP2 requires homo-dimerization or dimerization with paralogs FOXP1 or FOXP4. Whether FoxP dimerization occurs in the brain is unknown. We recently showed that FoxP1, FoxP2 and FoxP4 (FoxP1/2/4) proteins are co-expressed in neurons of Area X, a song control region in zebra finches. We now report on dimer- and oligomerization of zebra finch FoxPs and how this affects transcription. In cell lines and in the brain we identify homo- and hetero-dimers, and an oligomer composed of FoxP1/2/4. We further show that FoxP1/2 but not FoxP4 bind to the regulatory region of the target gene Contactin-associated protein-like 2 (*CNTNAP2*). In addition, we demonstrate that FoxP1/4 bind to the regulatory region of very low density lipoprotein receptor (*VLDLR*), as has been shown for FoxP2 previously. Interestingly, FoxP1/2/4 individually or in combinations regulate the promoters for SV40, zebra finch *VLDLR* and *CNTNAP2* differentially. These data exemplify the potential for complex transcriptional regulation of FoxP1/2/4, highlighting the need for future functional studies dissecting their differential regulation in the brain.

## Introduction

Forkhead box (Fox) transcription factors comprise 19 highly evolutionary conserved, structurally related families, FoxA to FoxS. The defining feature of these genes is the Fox domain which binds to regulatory regions of target genes. Many *Fox* genes perform tissue specific functions during development and mutations can cause cancer and other diseases (Hannenhalli and Kaestner, 2009).

The FoxP family consists of only one member in invertebrates (Santos et al., 2011). Gene duplication gave rise to four *FoxP* subfamily members in vertebrates, *FoxP1* to *FoxP4* (Song et al., 2016). Expression of these four proteins is specific to particular organs and cell types, with partly overlapping patterns (Lu et al., 2002; Ferland et al., 2003; Mendoza et al., 2015; Spaeth et al., 2015). FoxP1/2/4 are all expressed in the brain (Lu et al., 2002; Teufel et al., 2003), whereas FoxP3 is prominently expressed in T regulatory cells of the immune system (Huehn et al., 2009).

In humans, *FOXP1* and *FOXP2* mutations impair speech production and perception (Bacon and Rappold, 2012). FoxP1 has also been linked to autism spectrum disorder (ASD; Girirajan et al., 2011; Bowers and Konopka, 2012). A human FOXP4 mutation was associated with developmental delay, heart and larynx problems (Charng et al., 2016).

We study FoxP proteins in songbirds because birdsong and speech share many features (Doupe and Kuhl, 1999). Humans and songbirds learn a large fraction of the sounds they use to communicate through auditory-guided vocal imitation. Vocal production learning of speech and birdsong is constrained by innate predispositions, speech and song learning is best achieved during critical developmental periods and strongly affected by social factors. Birdsong and speech depend on analogous neural pathways that are functionally lateralized (Petkov and Jarvis, 2012; Pfenning et al., 2014). Because of the many parallels between the development of birdsong and speech, songbirds provide a genuine model for behavioral, neural and molecular analyses of genes in the context of vocal communication (Bolhuis et al., 2010). Temporally and spatially precise manipulations of FoxP2 amounts in striatal nucleus Area X, a basal ganglia component of the neural circuit controlling song production and song learning, results in incomplete and inaccurate vocal imitation, alters adult song production, spine density and neural transmission (Haesler et al., 2007; Schulz et al., 2010; Murugan et al., 2013; Heston and White, 2015). The impact of FoxP1 and FoxP4 manipulations on song learning has not been reported, but both FoxPs can co-occur with FoxP2 in the medium spiny neurons (MSNs) of Area X (Mendoza et al., 2015).

Mice with homozygous deletions of *Foxp1, Foxp2* alone or in combination, or of *Foxp4*, die before or shortly after birth (Li et al., 2004b; Wang et al., 2004; Shu et al., 2005, 2007; Rousso et al., 2012). Heterozygous mutations in mice are associated with deficits in synaptic function, motor behaviors (Groszer et al., 2008; French and Fisher, 2014; Fröhlich et al., 2017) and impact the development and adult production of ultrasonic vocalizations (Castellucci et al., 2016; Chabout et al., 2016). *Foxp4* mouse mutants have numerous brain and spinal cord defects (Rousso et al., 2012).

*Drosophila melanogaster* with FoxP mutations or with RNAi mediated manipulations of *FoxP* expression exhibit deficits in an odor-based decision paradigm (DasGupta et al., 2014), in motor coordination and courtship song (Lawton et al., 2014), and in operant self learning (Mendoza et al., 2014).

Among the Fox family of transcription factors, the members of the P subfamily are unique in their requirement to bind to another FoxP protein for transcriptional regulation. Both homo- and hetero-dimerization can occur, mediated by two evolutionary conserved protein domains, the zinc-finger and leucine-zipper (Wang et al., 2003; Li et al., 2004a; Mozzi et al., 2016). A recent study reported episodic positive selection around the leucine-zipper of *FoxP2* in specific avian lineages with possible consequences for dimerization (Mozzi et al., 2016). Dimerization of FoxP proteins has so far only been assessed by overexpressing the mouse (Li et al., 2004a) and human (Sin et al., 2015) protein versions in cell lines. The relevance of FoxP protein-protein interaction is emphasized by the fact that *FOXP3* mutations in the dimerization domain cause IPEX syndrome (Immune dysregulation, polyendocrinopathy, enteropathy, X-linked human syndrome; Li et al., 2007). Furthermore, a polymicrogyria patient with a mutation in the leucine zipper region of *FOXP2* showed dysregulation of one of its target genes, *SRXP2* (Roll et al., 2010).

Despite the fact that FoxP factors have the capacity to dimerize in cell lines (Li et al., 2004a; Sin et al., 2015), it is not known whether this interaction also takes place in the vertebrate brain. Overlapping expression of two or more FoxP members occur in the brain of various vertebrates (Teramitsu et al., 2004; Takahashi et al., 2008; Rodenas-Cuadrado et al., 2014; Bowers et al., 2014; Whitney et al., 2015) but few studies have analyzed co-expression at single cell resolution (Bowers et al., 2014; Mendoza et al., 2015; Whitney et al., 2015). In mice, Foxp2 and Foxp4 are co-expressed in spinal cord motor neuroblasts, and the quantity of Foxp4 protein expressed in those neurons is important for their differentiation (Rousso et al., 2012). In the rat the majority of MSNs co-express Foxp1/2 (Bowers et al., 2014). In the zebra finch FoxP1/2/4, are expressed in specific brain regions with different degrees of overlap (Mendoza et al., 2015). In Area X, FoxP1/2/4 are co-expressed in a large fraction of striatal MSN, but all other combinations of co-expression also exist to different extents in this cell type (Mendoza et al., 2015). In budgerigar birds FoxP1/2 also co-localize in the majority of striatal MSN (Whitney et al., 2015). These findings indicate that interactions among the FoxP proteins are possible but do not show that they actually take place.

The aim of this study was to assess whether FoxP1/2/4 of the zebra finch can dimerize in cell lines and in the brain. Furthermore, we asked whether FoxP1 and FoxP4 are able to bind to regulatory regions of two neurally relevant genes, very low density lipoprotein receptor (*VLDLR*), encoding one of the reelin receptors, and Contactin-associated protein-like 2 (*CNTNAP2*) gene which codes for a neurexin called CASPR2 (Rodenas-Cuadrado et al., 2014). Both proteins were previously recognized to be regulated by FoxP2 (Spiteri et al., 2007; Vernes et al., 2007, 2008; Adam et al., 2016). Finally we survey the transcriptional regulation of FoxPs expressed individually or in combination, to explore whether homo-dimers and hetero-dimers fulfill different functions. Our results provide the first evidence for molecular interactions of FoxP subfamily members in the brain. We also show that different combinations of FoxP proteins regulate target genes differentially. These findings underscore the need to take these interactions into account in future studies that address why FOXP1 and FOXP2 mutations are associated with the development of impaired speech and other diseases.

## Materials and Methods

### Subjects

Male zebra finches (*Taeniopygia guttata*) were bred in our colony at the Freie Universität Berlin. All procedures were performed according to the guidelines of the governmental law (TierSchG), under permits granted by the local Berlin authorities governing research involving animals. All birds were sacrificed with an Isoflurane overdose.

### Co-Immunoprecipitation (Co-IP)

Co-Immunoprecipitation (Co-IP) was done using Dynabeads® Protein G (Invitrogen, Cat.No.100.04D) following the manufacturer’s protocol with a few changes as follows. We first incubated the antibody-Dynabeads mixture for 15 min at room temperature (for antibody concentrations refer to Table 1). We subsequently incubated the protein extracts plus antibody-coated Dynabeads mixture for 30 min at 4°C with rotation. We eluted with 20 μl of elution buffer (50 mM Glycin pH 2.8) and before a second round of immunoprecipitation, we neutralized the elution buffer by adding 4.8 μl 1 M TrisHCl pH 7.5 and then added washing buffer to bring the volume to a total of 200 μl. After elution we added 15 μl of 2× Laemmli and prepared lysates for denaturing conditions.

**Table 1:** **Antibodies**.

Antigen	Immunogen	Manufacturer, species raised in, mono/polyclonal, Cat. no.	Dilutions
Beta-actin	A slightly modified synthetic b-cytoplasmic actin N-terminal peptide DDDIAALVIDNGSGK conjugated to KLH	Sigma, mouse monoclonal, A5441	1:250000-500000 Western blot
FLAG-M2	DYKDDDDK FLAG epitope	Stratagene (Agilent), mouse monoclonal, 200471	1:2000-10000 Western blot/8 μg CoIP
Myc	EQKLISEEDL tag human c-Myc, AA 410-419	Roth, rabbit polyclonal, 4667.1	1:2000-10000 Western blot/8 μg CoIP
V5	GKPIPNPLLGLDST	Life Technologies, mouse monoclonal, R960-25	1:2000-10000 Western blot
FoxP1	Full length native protein, purified from mouse FOXP1	Abcam, mouse monoclonal, ab32010	1:2000-5000 Western blot/8 μg CoIP
FoxP2	Synthetic peptide (C) REIEEEPLSEDLE corresponding to amino acids 703-715 of the C-terminus of human FOXP2	Abcam, rabbit polyclonal, ab16046	1:2000-5000 Western blot/8 μg Co-IP
FoxP4	The epitope recognized by A302-394A maps to a region between residue 1 and 50 of human forkheadbox P4 using the numbering given in entry NP_001012426.1.	Bethyl, rabbit polyclonal, A302-394A	1:2000-5000 Western blot/8 μg Co-IP

### Western Blots and Detection

Transfected HeK293 cells were treated with M-PER (Thermo Scientific, Prod#78505) lysis medium for 15 min on ice. Extracts were centrifuged for 10 min at 1500 g and supernatant was stored at −80°C until used. Cell extracts and co-IP’ed proteins were separated by SDS PAGE (8%–10%), transferred to a polyvinylidene fluoride membrane (Roche, Indianapolis, IN, USA), and blocked with Roti-Immunoblock for 2 h or overnight at 4°C. The membranes were then incubated with the desired antibody (Table 1) overnight at 4°C. Membranes were subsequently washed 3× PBS/0.1% Tween 20 followed by incubation with an HRP-conjugated antibody raised in the appropriate animal (1:2000 dilution; Amersham Biosciences) for another 30 min. Binding was detected on X-ray films using an ECL detection system for HRP (Perkin-Elmer, Boston, MA, USA). Films were developed in a Curix 60 developing machine (Agfa, Cologne, Germany). After the first detection membranes were washed in PBS/0.1% Tween for 5 min, then washed in 0.5 NaOH for 10 min, washed again in PBS/0.1% Tween for 5 min and blocked again and detected with a second/third primary antibody as described before.

### Brain Dissection and Microbiopsies

After sacrificing the bird, the forebrain was quickly dissected, the hemispheres separated, embedded in TissueTec and frozen on dry ice. Hemispheres were stored at −80°C until further processing. Area X microbiopsies were punched as described previously (Olias et al., 2014; Adam et al., 2016). Protein was extracted from pooled microbiopsies of the same animal after we had confirmed correct targeting of the desired brain region (Olias et al., 2014; Adam et al., 2016).

### Luciferase Promoter Reporter Assay

For SV40 we seeded ~30000 HeLa cells in 200 μl DMEM medium (GIBCO) containing Penicillin-Streptomycin (Lonza-DE17–602E, 10 UI/ml) per well of a 96-well white flat bottom plate (Nunclon, Cat.No.136101, Denmark). Transfection was performed using Lipofectamine™ 2000 (Invitrogen) following manufacturer’s protocol. Briefly, after seeding plates were incubated for 24 h at 37°C at 5% CO_2_. For transfection, the medium was exchanged with 100 μl of antibiotic free medium before adding the 50 μl transfection mix in each well. Transfection mix consisted of two parts: (a) 25 μl of OptiMEM (GIBCO) containing 30 ng of pGL4.13 (Luciferase gene driven by the SV40 promoter which is known to be regulated by FoxP subfamily members) and 30 ng of pGL4.75 (Renilla gene driven by the CMV promoter that is not affected by FoxP subfamily members, used for normalization of expression changes) and 250 ng total vector over-expressing the different FoxPs for each well; and (b) 1 μl of Lipofectamine™ 2000 (Invitrogen) in 25 μl OptiMEM that was premixed for 5 min at RT. After combining (a) and (b) the mix was incubated for 20 min at RT, then it was added to the cells. After 4–6 h of incubation we changed the medium to 75 μl of antibiotic containing medium and incubated for further 48 h at 37°C in a CO_2_ incubator.

For *VLDLR* and *CNTNAP2* we used HeK293 cells, and luciferase assays were done as described previously (Adam et al., 2016). Depending on the promoter, we performed between 4 and 7 luciferase assays, each after an independent transfection. Within each assay we used triplicates, e.g., three wells containing the same transfection reagents and quantity of cells. The mean of the triplicate was used for statistical analysis. Each plate was measured once in the ELISA reader.

We measured luminescence using the Dual Glo Luciferase Kit (Promega) following manufacturer’s protocol in an Elisa plate reader (Tecan, GENios; Switzerland). Mean background from untransfected wells was subtracted from all other wells. We present Luciferase results as mean Relative Light Units (Luciferase RLU/Renilla RLU) calculated from the normalized values of 4–7 independent assays.

### Cloning of VLDLR and CNTNAP2 Promoters

The pGL4-VLDLR promoter was cloned as described (Adam et al., 2016). The CNTNAP2 promoter was cloned using DNA obtained from a blood sample of an adult bird as the template and amplified with the forward primer 5′-TTGCCTCATTGATTGCAGAA-3′ and reverse primer 5′-CCTGCTTTTCTCCACTTTGG-3′ using High Fidelity Taq (Fermentas K0191). The resulting PCR product was examined on an agarose gel, cleaned from nucleotides with the Nucleo Spin Gel and PCR Clean-up (Macherey-Nagel, Germany, Ref 740609.250), and cloned into the pCR4Blunt-TOPO vector of the Zero blunt PCR cloning kit (Invitrogen) according to the manufacturer’s protocol. Inserts from three independent CNTNAP2 clones were fully sequenced to confirm the sequence of the promoter region of CNTNAP2. Zebra finch CNTNAP2 sequences were deposited to GenBank, accession number NCBI KX943238. We then used forward primer 5′-GATGCTAGCTTGCCTCATTGATTGCAGAA-3′ and reverse primer 5′-GATAGGCCTCCTGCTTTTCTCCACTTTGG-3′ and subcloned the PCR product into the NheI and StuI sites of the pGL4.13 vector to generate the pGL4-CNTNAP2 vector used in Luciferase assays.

We identified putative CpG islands in the CNTNAP2 promoter with the CpG plot tool^2^. We calculated the GC percentage in the GC rich region of the promoter region of CNTNAP2 using the CpG island calculator^3^.

### Overexpression and Transfection of HeK293 Cells

Overexpression vectors of FoxP1/2/4 tagged with FLAG or V5 were previously generated (Haesler et al., 2004; Mendoza et al., 2015). For the generation of Myc tagged FoxP2 we used the FoxP2-V5 tagged sequence as a template and used the forward primer 5′-CGCGGATCCGCCACCATGATGCAGGAATCTGCGACAG-3′ and reverse primer 5′-GCGGAATTCCTACAGATCCTCTTCTGAGATGAGTTTTTGTTCTTCCAGATCTTCAGATAAAGGCTC-3′ and cloned it into pcDNA3, 1 + vector (Invitrogen).

### Electrophoretic Mobility Shift Assays (EMSA)

Proteins for electrophoretic mobility shift assays (EMSAs) were purified using the Protino Ni-NTA Agarose (Macherey-Nagel, Germany, 745400.25) according to manufacturer’s protocol. Protein was quantified using BCA1 (Sigma). EMSA assays were carried out as published previously, using the Oligo described for VLDLR (Adam et al., 2016). The oligo we used for CNTNAP2 was 5′-TATTAT**TATTTATTTTT**GTACTCTACATTCCTTGT**TATTTGAT**ACT-3′ (in bold presumed FoxP binding sites containing the ATTT core sequence).

### Statistical Analysis

Statistical tests were performed using the data analysis software R (R Core Team, 2013). After testing for normality, differences between Luciferase experiments were calculated with an analysis of variance (ANOVA) and a Tukey’s HSD *post hoc* test for pairwise comparison (R Core Team, 2013). Graphs were prepared with GraphPad Prism 4.0 (GraphPad Software, San Diego, CA, USA). Data are expressed as mean of means ± SEM.

## Results

### FoxP1/2/4 Zebra Finch Proteins Homo- and Hetero-Dimerize in Cell Lines

**Figure 1:**
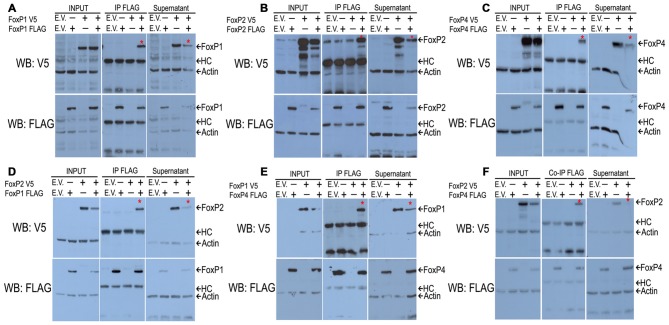
**Western blots after co-immunoprecipitations show that all FoxP1/2/4 zebra finch proteins can homodimerize (A–C)** and heterodimerize **(D–F)** *in vitro*. HeK293 cells were transfected with combinations of expression vectors encoding FoxP1/2/4 proteins that were tagged with FLAG or V5. The FLAG monoclonal antibody was used to immunoprecipitate proteins from cell extracts. Immunoprecipitated proteins were resolved on SDS-polyacrylamide gels, transferred to nitrocellulose and analyzed by sequential immunoblotting with V5 and FLAG antibodies. In all co-immunoprecipitations **(A–F)**, from left to the right, the first lane shows the empty vector (E.V.) as a negative control; lanes 2 and 3 show protein extracts of transfections with only one of the two tagged proteins as further controls; lane 4 shows the protein extract from transfection with both proteins. The upper panels show the V5 detection, and the lower panels the subsequent FLAG detection. For all conditions and detections we show the input proteins, co-immunoprecipitated proteins and the supernatant after co-immunoprecipitation. In all cases, there is a V5 protein co-immunoprecipitated with the FLAG antibody in the lane where both proteins are present and a reduction of the co-immunoprecipitated protein in the supernatant, showing an interaction of both proteins marked with an asterisk (*).

To determine whether zebra finch FoxP1/2/4 can homo- and hetero-dimerize in cell lines, as described for the mouse FoxPs (Li et al., 2004a) and human FOXPs (Sin et al., 2015), we transiently overexpressed full length constructs of zebra finch FoxPs tagged either with FLAG or V5 in HeK293 cells and performed co-immunoprecipitation assays. In each Co-IP experiment we used four protein lysates; empty vector lysate (control 1), lysate with one of the two proteins to be tested for co-immunoprecipitation (control 2), another lysate with the other protein to be tested (control 3), and the lysate containing both proteins (experimental). Lysates were co-IP’ed with FLAG antibody, and membranes were first detected with V5 antibodies and sub-sequentially detected with FLAG antibodies. FLAG tagged proteins were immunoprecipitated as expected from lysates containing FLAG-tagged proteins whereas this was not the case for cells expressing an empty vector, or from cell lysates containing V5 tagged proteins (Figures 1A–F). In all experiments we detected a protein co-immunoprecipitated in lysates co-transfected with two differently tagged FoxPs (Figures 1A–F), indicating protein-protein interactions of the co-expressed FoxPs. Lastly, in all cases the supernatant was mostly depleted of the co-IP’ed protein (Figures 1A–F). From these results we conclude that zebra finch FoxP1/2/4 homologs are able to form homo- (Figures 1A–C) and hetero-dimers (Figures 1D–F) in cell lines.

### FoxP1/2/4 Antibodies Are Specific

**Figure 2:**
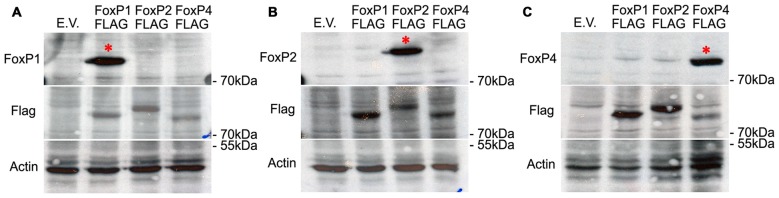
**Western blots demonstrating the specificity of the different FoxP antibodies by detecting only one FoxP protein in extracts of HeK293 cells transfected with an over-expression vector carrying either empty vector (E.V.), or the three FLAG-tagged FoxP proteins**. Proteins were resolved by SDS-PAGE, transferred to nitrocellulose, and first detected (top panels) with anti-FoxP1 **(A)**, or anti-FoxP2 **(B)**, or anti FoxP4 **(C)** and sequentially detected FLAG/b-actin antibodies as loading controls (middle panels) **(A–C)**. Bottom panels show detection of actin in the samples as a loading control. In all cases, the specificity of the antibody is evident from a single band in the expected lane (*).

To examine whether FoxP1/2/4 proteins also interact in neurons of the zebra finch forebrain, we first characterized commercial antibodies against FoxP1/2/4 zebra finch proteins by over-expressing them individually in HeK293 cells and performing Western Blots. Specific antibodies show a band of about 80 kDa molecular weight in each case. Each antibody only recognized one FoxP (Figures 2A–C, top panel), and not the other two. The faint bands in the FoxP1 and FoxP2 lanes in panel c do not correspond to cross reactivity of FoxP4 antibody against FoxP1 and FoxP2 protein, which have a slightly different molecular weight, as can be seen in the FLAG tagged versions (middle panel). The subsequent detection with FLAG (and actin antibodies, lower panel) also demonstrates that over-expressed proteins were present in the protein lysate in similar quantity (Figures 2A–C). From these results we conclude that antibodies are specific for the different zebra finch FoxPs.

### FoxP1/2/4 Zebra Finch Protein Hetero-Dimerize in the Brain

**Figure 3:**
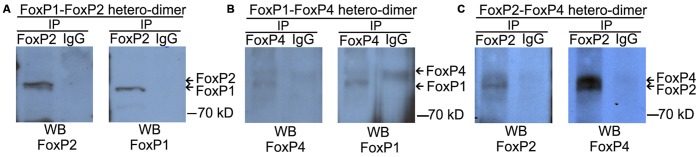
**FoxP1/2/4 can hetero-dimerize in the brain**. The three panels depict representative co-immunoprecipitation experiments from nuclear protein extracts of adult zebra finch brains with anti-FoxP2 **(A,C)**, anti-FoxP4 **(B)**, or nonspecific IgG **(A–C)** under non-denaturing conditions. Proteins were resolved by SDS-PAGE, transferred to nitrocellulose, and analyzed by sequential immunoblotting with anti-FoxP1 (**A**-left and **B**-right, Westerns), or anti-FoxP2 (**A,C** left Westerns), or anti FoxP4 (**B,C** right panels). In all cases, a hetero-dimer was co-immunoprecipitated with the specific antibodies from whole forebrain lysate and no signal of the same size was detected in the IgG control, suggesting an interaction of FoxP proteins.

We performed co-IP assays on protein lysates of adult male zebra finch forebrain using the specific antibodies described above. FoxP1 monoclonal antibody was not able to pull down the non-denatured, native brain protein (data not shown), and therefore we used FoxP2 and FoxP4 polyclonal antibodies to pull down protein complexes, and all three antibodies for Western blot detection. As a negative control we used IgG antibodies of the same species in which the specific FoxP antibodies were raised. After IP with FoxP2 or FoxP4 antibodies, we detected co-IP’ed FoxP1 (Figures 3A,B). After IP with FoxP2 we detected co-IP’ed FoxP4 (Figure 3C). In contrast, no protein of the expected size was precipitated with IgG controls (Figures 3A–C). From these results we conclude that FoxP1/2/4 form hetero-dimers in forebrain neurons *in vivo*. To our knowledge this is the first report showing that FoxP1/2/4 proteins can form hetero-dimers in the brain.

### FoxP1/2/4 Zebra Finch Proteins form an Oligomer in a Cell Line

**Figure 4:**
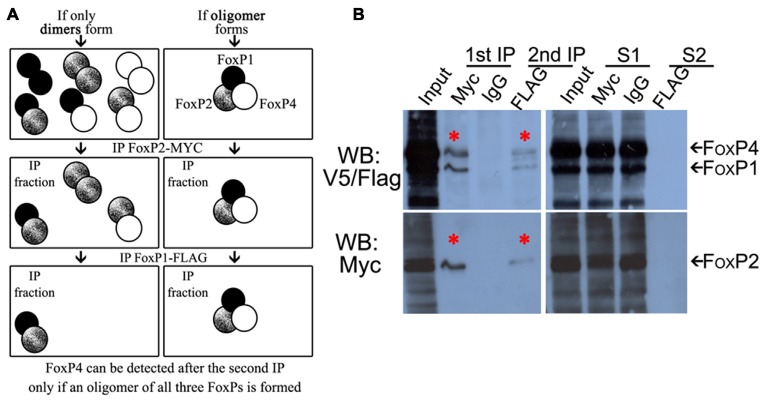
**FoxP1/2/4 zebra finch proteins can oligomerize** *in vitro***. (A)** Schematic representation of possible combinations of dimers (left panel) or a multimer (right panel) of FoxP1/2/4 in IPs. **(B)** Western blots of double-immunoprecipitation of HeK293 cells transfected with combinations of expression vectors encoding FoxP1 FLAG-tagged, FoxP2 Myc-tagged, and FoxP4 V5-tagged proteins, revealing a FoxP1/2/4 multimer. The left panel of the Western blot shows, from left to right, in the first lane the input protein extract after transfection with all three proteins, followed by lane 2 showing the immunoprecipitated FoxP2-Myc (lower panel, asterisk) with the co-immunoprecipitated FoxP1-FLAG and FoxP4-V5 (upper panel, asterisk). Lane 3 shows the absence of immunoprecipitated proteins when using IgG (rabbit), lane 4 shows the subsequent immunoprecipitation of FoxP1-FLAG (upper panel, asterisk) and co-immunprecipitated FoxP4 and FoxP2 (upper and lower panels respectively, asterisks). In the right Western blot the first lane is again the input, followed by the supernatant of the first immunoprecipitation with Myc and IgG rabbit in lanes 2 and 3, and then the supernatant of the subsequent immunoprecipitation with FLAG.

Since the majority of FoxP expressing cells in Area X and the surrounding striatum express FoxP1/2/4 (Mendoza et al., 2015), and since it is known that FOXP3 is able to homo-oligomerize and hetero-associate in cell lines (Li et al., 2007; Song et al., 2012), we wanted to assess whether all three neurally expressed FoxP proteins could bind in a single protein complex. To test this we performed double co-IP from HeK293 cell lysates co-transfected with full length FoxP1-FLAG, FoxP2-Myc and FoxP4-V5 tagged zebra finch proteins (Figure 4A schematic). If FoxP1/2/4 were not able to hetero-associate in a complex after double co-IP we should only detect the two proteins that were immunoprecipitated, but if they hetero-associate in a complex we should be able to detect all three FoxPs. We first immunoprecipitated with Myc, to pull down FoxP2 and all proteins bound to it. After the first immunoprecipitation we found that all three proteins had co-IP’ed (Figure 4B). The fact that we detected FoxP1-FLAG and FoxP4-V5 after immunoprecipitating the Myc-taggd FoxP2 could be due to pulled down hetero-dimers of FoxP2 with FoxP1 or with FoxP4. Alternatively, FoxP1/2/4 could have been simultaneously pulled down by immunoprecipitating Myc-tagged FoxP2. To assess this, a second, sequential IP with FLAG was carried out to detect FoxP1-FLAG after the first IP. After the second immunoprecipitation we detected not only FoxP1-FLAG but also co-IP’ed FoxP4-V5 (Figure 4, upper blot).

Not all protein was depleted from the first supernatant, one possible explanation being that FoxP1 homodimers and FoxP4 homodimers were not precipitated by Myc antibodies detecting FoxP2-Myc. Leftover FoxP2 in the supernatant additionally indicates that not all FoxP2 was precipitated by the Myc antibody, likely because of insufficient antibody concentration to deplete all FoxP2 protein and its associated interaction partners. No protein was detected after the second immunoprecipitation in the second supernatant (Figure 4, S2).

In summary, these data suggest that FoxP1/2/4 can form a complex including all three proteins, a result which has not been reported before. In addition to this hetero-oligomerization, we also found homo- and hetero-dimerization of all FoxPs (Figures 1, 3).

### FoxP1/2/4 Zebra Finch Proteins Oligomerize *In Vivo*

**Figure 5:**
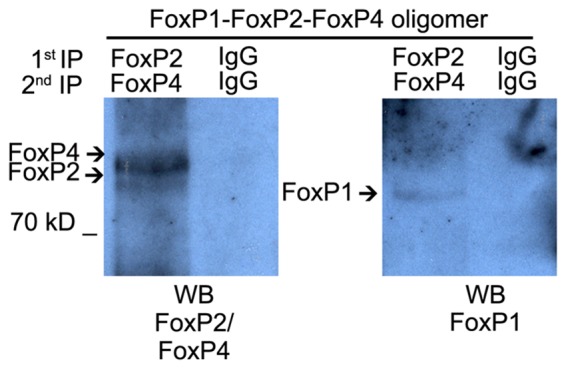
**Hetero-oligomerization of FoxP1/2/4 occurs in the brain, in song nucleus Area X**. Co-immunoprecipitation performed on nuclear protein extracts from microbiopsies of Area X. The protein extract was split into equal amounts. One was immunoprecipitated with FoxP2 antibody and subsequentially with FoxP4 antibody, the other half was immunoprecipitated with rabbit IgG two times sequentially. Immunoprecipitated proteins were resolved on SDS-polyacrylamide gels, transferred to nitrocellulose, and analyzed by sequential immunoblotting with FoxP2 and FoxP4 or FoxP1 antibodies.

To assess whether FoxP1/2/4 can hetero-oligomerize in the zebra finch song system we used nuclear protein extracts of Area X. Double IP using specific antibodies detected the three proteins in the co-immunoprecipitated fraction suggesting that FoxP1/2/4 can also hetero-associate *in vivo* in Area X (Figure 5).

### FoxP1/2/4 Zebra Finch Proteins Repress the SV40 Promoter in Luciferase Promoter Reporter Assays

**Figure 6:**
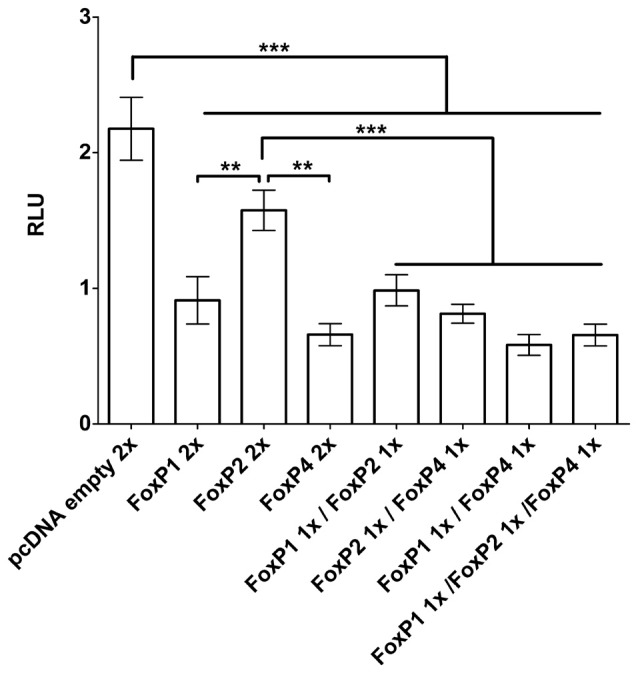
**Luciferase assays demonstrate transactivation properties of different combinations of FoxP1/2/4 proteins on the SV40 promoter**. FoxP1/2/4 as well as their combinations significantly repressed the pGL4.13-promoter transcriptional activity through a specific DNA-binding site in the SV40 promoter. Significance levels from all combinations to the empty vector control are represented by stars, **p < 0.001–0.01; ***p < 0.001. One way ANOVA; *F* = 28.79; DF = 7; *n* = 8; followed by Tukey’s multiple comparison test; Bars show mean of means ± SEM of five independent transfections for each of the eight conditions, presented as luciferase/renilla ratio (RLU), corrected for transfection by pGL4.75 Renilla luciferase activity. 1x = 125 ng of overexpressing vector per well, 2x = 250 ng of overexpressing vector per well. The control transfection value was obtained with the empty expression vector (pcDNA3.1).

We assessed whether the homotypic and heterotypic interaction of zebra finch FoxP proteins lead to differential regulatory activity. To do so we used the SV40 promoter driving Luciferase (*Photinus pyralis* synthetic protein). The SV40 promoter has a putative core consensus sequence for binding (T**ATTT**RT) human and murine Foxp1/2/4 (Wang et al., 2003; Vernes et al., 2006). For the human and mouse proteins it was shown in luciferase promoter reporter assays that each FoxP can repress the SV40 promoter individually (Wang et al., 2003; Li et al., 2004a; Vernes et al., 2006). Likewise, we found that zebra finch FoxP proteins when expressed individually in HeLa cells significantly repressed the transcriptional activity under the control of the SV40 promoter (Figure 6; One way ANOVA; *F* = 28.79; DF = 7; *n* = 8; *p* < 0.0001; Tukey’s Multiple comparison Test; compared to empty vector: FoxP1 *p* < 0.0001, FoxP2 *p* = 0.013, and FoxP4 *p* < 0.0001), in the same range as reported for mice and human FOXP1/2/4. Also, like its mammalian homologs, zebra finch FoxP4 repressed transcription stronger than FoxP1 or FoxP2 did, and FoxP2 was a weak repressor of the SV40 promoter (Wang et al., 2003; Li et al., 2004a; Vernes et al., 2006). Cells expressing combinations of the different FoxP subfamily members also showed a significant repression of the SV40 promoter (compared to empty vector: FoxP1/2 *p* < 0.0001; FoxP1/4 *p* < 0.0001; FoxP2/4 *p* < 0.0001 and FoxP1/2/4 *p* = 0.0001). There were no significant differences between cells expressing FoxP1 or FoxP4 alone or in any of the combinations tested. We did observe a stronger transcriptional repression in cells expressing FoxP2 in combination with another FoxP subfamily member than when FoxP2 was expressed alone (compared to FoxP2 alone: FoxP1/2 *p* = 0.016; FoxP2/4 *p* = 0.0005 and FoxP1/2/4 *p* < 0.0001). However, we cannot rule out the formation and action of homodimers of FoxPs in those cells. Taken together, FoxP target genes in cells that express only FoxP2 may be less strongly regulated than those that are co-expressed with FoxP1 and/or FoxP4.

### *VLDLR* Is a Direct Target Not Only of FoxP2 but Also of FoxP1/4

**Figure 7:**
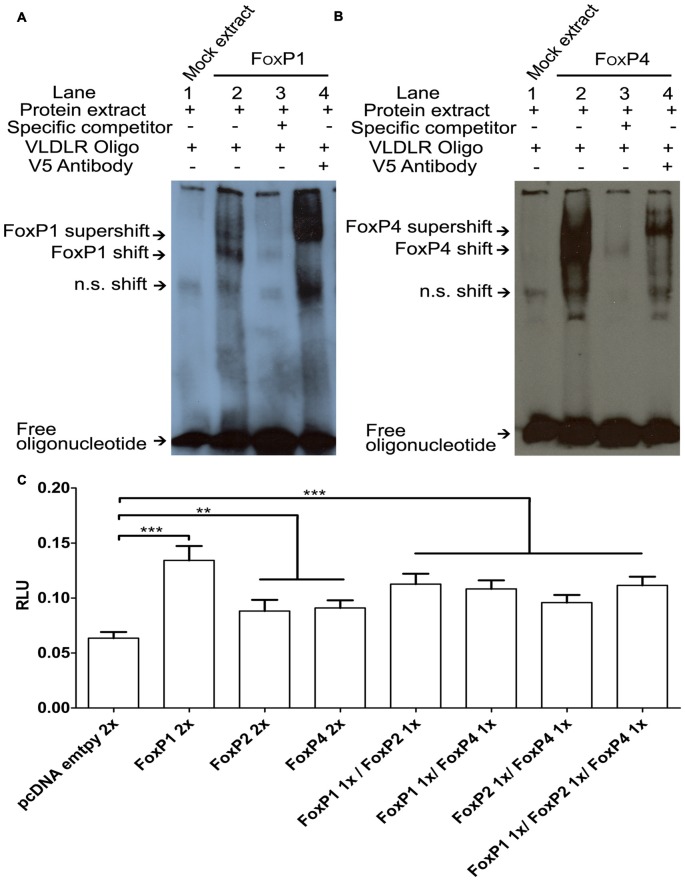
**FoxP1/2/4 bind and activate the very low density lipoprotein receptor** *(VLDLR)*** promoter**. DNA binding assays with FoxP1 **(A)** and FoxP4 **(B)**. Nuclear extracts (1 μg) from HeK293 cells were incubated with the digoxigenin labeled probe (0.8 ng) representing the 27-bp of the *VLDLR* FoxP2 binding site. Shown in each case are protein lysate of HeK293 cells transiently transfected with empty vector and labeled probe (lane 1), shift in the presence of nuclear extract of FoxPs (lane 2), and complex formation in the presence of 200-fold molar excess of specific un-labeled probe (lane 3) and supershift in the presence of labeled probe, FoxPs protein extract and monoclonal V5 antibody (1 mg/ml; lane 4). In all cases arrows point to free oligo, non specific shift (n.s.), FoxP shift and supershift. **(C)** Luciferase assays were carried out in HeK293 cells to measure effects of a FoxP1/2/4 alone or in combinations on the *VLDLR* promoter. Significance levels from all combinations to the empty vector control are represented by asterisk, **p < 0.001–0.01; ***p < 0.001. One way analysis of variance (ANOVA); *F* = 26.09; DF = 7 and *n* = 8; followed by Tukey’s multiple comparison. Bars show mean of means ± SEM of four independent transfections, presented as luciferase/renilla ratio (RLU), corrected for transfection by pGL4.75 Renilla luciferase activity. 1x = 125 ng of overexpressing vector per well, 2x = 250 ng of overexpressing vector per well. The control transfection value was obtained with the empty expression vector (pcDNA3.1).

To explore whether FoxP proteins not only regulate SV40 but also neurally relevant promoters we chose *VLDLR* for a number of reasons: (a) it is a direct target of FoxP2 in humans, mice, and zebra finches (Spiteri et al., 2007; Vernes et al., 2007; Adam et al., 2016); (b) both FoxP2 and VLDLR promote dendrite and dendritic spine development in various species (Niu et al., 2004, 2008; Schulz et al., 2010; Vernes et al., 2011); and (c) in songbirds, FoxP2 and VLDLR are expressed in Area X and the basal ganglia (Balthazart et al., 2008; Hilliard et al., 2012) and are co-regulated in different contexts (singing, age and by molecular manipulations; Adam et al., 2016). Most of the work on target genes in the FoxP subfamily that are expressed in the brain have focused on FoxP2, and it is not known how much overlap there is in the binding sites for FoxP1 and FoxP4 and whether they can bind to the same promoter motives. To investigate this we used the *VLDLR*-oligonucleotide that FoxP2 can bind to in EMSA (Adam et al., 2016) and tested whether FoxP1/4 also binds to this probe. The protein extract of HeK293 cells transfected with empty vector (mock extract) presented a non specific shift (n.s.; Figures 7A,B, first lane). In the presence of FoxP1 or FoxP4 a specific shift of the DNA was observed, indicating that both proteins are able to bind to the *VLDLR* oligonucleotide (Figure 7A,B, second lane). Addition of a specific competitor diminished the intensity of this band (Figure 7A,B, third lane). Adding a V5 antibody to the FoxP/oligonucleotide mix resulted in a further shift, strengthening the notion that that FoxP1 or FoxP4 generated the shift (Figure 7A,B, fourth lane). From these results we conclude that FoxP1 and FoxP4 can bind to the same *VLDLR* region that FoxP2 binds to, suggesting that all three FoxPs share a common target gene.

### FoxP1/2/4 Differentially Activate the *VLDLR* Promoter in Luciferase Promoter Reporter Assays

To test whether binding of FoxP1/2/4 to the *VLDLR* promoter leads to an alteration of transcription, we performed luciferase assays using the pGL4-VLDLR vector described previously (Adam et al., 2016). There were significant differences in activation of the *VLDLR* promoter depending on the specific combinations of FoxP proteins (One way ANOVA; *F* = 26.09; DF = 7 and *n* = 8; *p* < 0.0001; Figure 7C). Tukey’s Multiple comparison Test revealed that when compared to empty vector, FoxP1 was a significantly better activator of the VLDLR promoter than FoxP2 or FoxP4 (FoxP1 *p* < 0.0001, FoxP2 *p* < 0.007 and FoxP4 *p* = 0.0024). However, FoxP2 and FoxP4 were less effective and not significantly different from each other in their ability to activate the VLDLR promoter (*p* = 0.999). Combinations of FoxP1/2/4 did activate the VLDLR promoter to a similar degree (compared to empty vector: FoxP1/2 *p* < 0.0001; FoxP1/4 *p* < 0.0001; FoxP2/4 *p* = 0.0003 and FoxP1/2/4 *p* < 0.0001). Comparing the activation of FoxP2 alone to the FoxP1/2 and FoxP1/2/4 combination (*p* = 0.0078 and *p* = 0.012 respectively) we found a significant difference on the VLDLR promoter but not with the FoxP2/4 combination (*p* = 0.88). There were no differences between the individual activation by FoxP4 and the combinations with FoxP1 (*p* = 0.10) or FoxP2 (*p* = 0.98), but there was a significant difference to the FoxP1/2/4 combination (*p* = 0.034). Taken together FoxP1/2/4 are not only repressors, but are able to bind to the VLDLR promoter and activate it differently depending on the co-expression with other FoxPs.

### Closing the Gap of the *CNTNAP2* Zebra Finch Promoter

To further test the regulatory ability of zebra finch FoxPs on a target gene relevant in Area X, we chose *CNTNAP2* because: (a) *CNTNAP2* is associated with speech disorders, as well as ASD, dyslexia and intellectual disability (Rodenas-Cuadrado et al., 2014) some of which are also associated with FoxP mutations (MacDermot et al., 2005; Vargha-Khadem et al., 2005; Shriberg et al., 2006; Zeesman et al., 2006; Lennon et al., 2007; Pariani et al., 2009; Vernes et al., 2009; Carr et al., 2010; Hamdan et al., 2010; Horn et al., 2010; Bowers and Konopka, 2012; Rice et al., 2012; Žilina et al., 2012; Chien et al., 2013; Le Fevre et al., 2013; Palumbo et al., 2013; Toma et al., 2013); (b) *CNTNAP2* is a direct target of Foxp2 in mice and of FOXP1 in humans (Vernes et al., 2007; O’Roak et al., 2011); (c) FoxP1/2/4 and CNTNAP2 are expressed in Area X and the basal ganglia in zebra finches (Haesler et al., 2004; Panaitof et al., 2010; Condro and White, 2014; Mendoza et al., 2015); and (d) FoxP2 binds to the *CNTNAP2* promoter region in zebra finches and the expression of both is correlated in adult undirected singing and in non-singing juveniles as well as after FoxP2 lentiviral knockdown (Adam et al., in review; personal communication).

**Figure 8:**
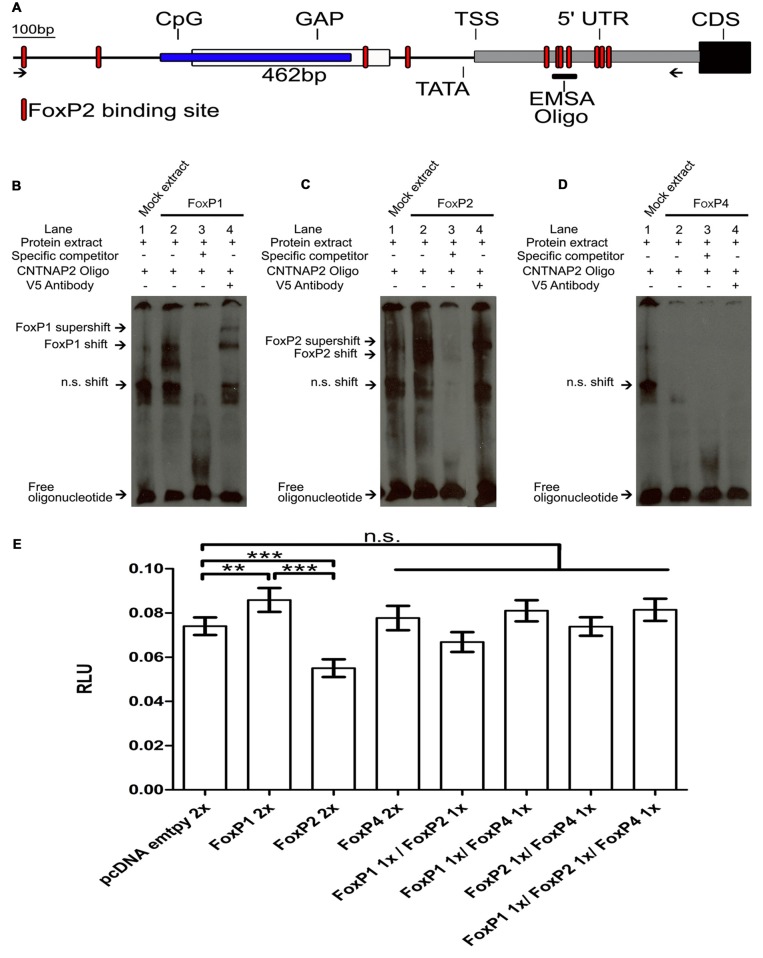
**FoxP1 activated, FoxP2 repressed and FoxP4 did not bind or regulate the Contactin-associated protein-like 2 (***CNTNAP2***) promoter. (A)** Schematic of the *CNTNAP2* promoter region. Arrows show the region of the primers used to clone the *CNTNAP2* promoter region. The location of the predicted transcription start site (TSS), TATA-box, CpG island (blue box), GAP (white box), 5′ UTR (gray box), coding sequence (CDS, black box) and FOXP2 binding sites (red shapes) are denoted by lines. The fragment used for the EMSA experiments (described in Adam et al., in review) is illustrated by the vertical line labeled “EMSA oligo” on the 5′ UTR region. DNA binding assays with FoxP1 **(B)**, FoxP2 **(C)** and FoxP4 **(D)** with the *CNTNAP2* oligo. Nuclear extracts (1 μg) from HeK293 cells were incubated with the digoxigenin labeled probe (0.8 ng) representing the 46-bp of the *CNTNAP2* FoxP2 binding site. Shown in each case are protein lysate of HeK293 cells transiently transfected with empty vector and labeled probe (lane 1), shift in the presence of nuclear extract of FoxPs (lane 2), and complex formation in the presence of 200-fold molar excess of specific un-labeled probe (lane 3) and supershift in the presence of labeled probe, FoxPs protein extract and monoclonal V5 antibody (1 mg/ml; lane 4). In all cases arrows point at free oligo, non specific shift (n.s.), FoxP shift and supershift. **(E)** Luciferase assays were carried out in HeK293 cells to measure effects of FoxP1/2/4 alone or in combinations on the *CNTNAP2* promoter. Significance levels from all combinations to the empty vector control are represented by asterisks, **p < 0.001–0.01; ***p < 0.001. One way ANOVA; *F* = 21.66; DF = 7 and *n* = 8; followed by a Tukey’s multiple comparison test. FoxP1 and FoxP2 single transfections significantly activated or repressed the pGL4-CNTNAP2 transcriptional activity through a specific DNA-binding site in the CNTNAP2 promoter (One way ANOVA; Tukey’s multiple comparison test; **P < 0.005; ***P < 0.0001). FoxP4 as well as all other combinations did not regulate the CNTNAP2 promoter. Bars show mean of means ± SEM of seven independent transfections presented as luciferase/renilla ratio (RLU), corrected for transfection by pGL4.75 Renilla luciferase activity. 1× = 125 ng of overexpressing vector pro well, 2× = 250 ng of overexpressing vector per well. The control transfection value was obtained with the empty expression vector (pcDNA3.1).

The assembled genome region of the zebra finch *CNTNAP2* promoter contained a sequence gap located ~0.75 kb upstream of the start codon of the first exon of *CNTNAP2* (Figure 8A). We cloned and sequenced across the gap, identifying a 462bp fragment, which had a high GC-content of up to 79.50% and was within the CpG island. The CpG island was located ~0.83–1.2 kb upstream of the star codon of the first exon. A TATA box was identified ~20 bp of the transcription start site (TSS). This region had 11 FoxP2 binding sites (Figure 8A, red shapes; Pierrou et al., 1994; Stroud et al., 2006; Nelson et al., 2013), four located in the promoter region and 7 in the 5′ UTR region. We were able to close the gap in the genome enabling us to further investigate the previously incomplete *CNTNAP2* promoter sequence and use it for luciferase assays.

### EMSA Reveals that FoxP1/2 Bind to the *CNTNAP2* Promoter but FoxP4 Does Not

We used an oligonucleotide probe from a sequence in the 5′ UTR region of the zebra finch *CNTNAP2* (Figure 8A) to test whether FoxP1/2/4 bind to it. This oligomer was identified in a parallel study on FoxP2 and *CNTNAP2* interactions interactions (Adam et al., in review). FoxP1 was able to bind to the *CNTNAP2* oligo and resulted in two shifted bands, consistent with the possibility that it binds in two of the three FoxP2 binding sites (Pierrou et al., 1994; Enard et al., 2009; Nelson et al., 2013) contained in the oligo (Figure 8B, “Materials and Methods” Section). We also confirmed the results of Adam et al. (in review), showing that FoxP2 also binds (Figure 8C). Interestingly, FoxP4 did not result in a DNA shift in the EMSA (Figure 8D).

### FoxP1 Activates, FoxP2 Represses and FoxP4 Does Not Regulate the *CNTNAP2* Promoter in Luciferase Promoter Reporter Assays

To investigate whether binding of FoxP1 and FoxP2 to the *CNTNAP2* promoter leads to an alteration of transcription, we performed luciferase assays using the pGL4-*CNTNAP2* vector (Figure 8E). When tested individually, FoxP1 activated the *CNTNAP2* promoter and FoxP2 repressed it. As expected from the lack of binding ability of FoxP4 to the CNTNAP2 promoter oligo (Figure 8D) FoxP4 failed to regulate the *CNTNAP2* promoter in the Luciferase assay (Figure 8E; One way ANOVA; *F* = 21.66; DF = 7 and *n* = 8; *p* < 0.0001; Tukey’s Multiple comparison Test; compared to empty vector: FoxP1 *p* = 0.005, FoxP2 *p* < 0.0001 and FoxP4 *p* = 0.91). Moreover, in combination, the different FoxPs also failed to regulate expression driven by the *CNTNAP2* promoter, (compared to empty vector: FoxP1/2 *p* = 0.26; FoxP1/4 *p* = 0.28; FoxP2/4 *p* = 1 and FoxP1/2/4 *p* = 0.22). Interestingly, combinations of FoxP4 with either FoxP1 or FoxP2 did not result in repression or activation, in contrast to FoxP1 or FoxP2 alone, suggesting that hetero-dimerization might affect the regulation of FoxPs. Taken together we show that FoxP1 and FoxP2 can bind to and regulate the *CNTNAP2* promoter, with opposing activities. *CNTNAP2* is an example of how FoxPs may tune the regulation of different targets genes via homo- and hetero-dimerization.

## Discussion

The present study demonstrates for the first time that FoxP proteins can not only dimerize in cell lines, as previously shown for mice (Li et al., 2004a) and humans (Sin et al., 2015), but also in the brain. Moreover, we discovered that FoxP proteins can also oligomerize, in cell lines and in the brain, also a novel finding. Specifically, we show that FoxP1/2/4 proteins can associate with each other in Area X, a song nucleus relevant for vocal learning, whose function depends on adequate FoxP2 protein amounts (Miller et al., 2008; Thompson et al., 2013). In addition, we compared the potential of the neurally expressed FoxP proteins to bind to the regulatory regions of *SV40*, *VLDLR* and *CNTNAP2* and regulate their transcriptional activity. Zebra finch FoxP1/2/4 repressed the *SV40* promoter activity, as reported for mouse and human (Wang et al., 2003; Vernes et al., 2006). In contrast, FoxP1/2/4 all activated the *VLDLR* promoter. Interestingly, the *CNTNAP2* promoter was regulated differentially; whereas FoxP1 activated it, FoxP2 repressed it and FoxP4 neither bound nor regulated this promoter. Together, these results emphasize the functional importance of the protein-protein interactions among the FoxP subfamily members in regulating their target genes. Since we previously showed that FoxP1/2/4 are expressed in different combinations in the MSN of the basal ganglia, including Area X (Mendoza et al., 2015), the present findings provide important steps toward understanding how the combinatorial regulation of FoxP2 with its interaction partners may regulate neural function in a circuit relevant for vocal production learning, such as speech in humans and song in birds.

Our findings that different combinations of FoxP1/2/4 can activate the promoter for the reelin receptor *VLDLR* with different strengths are likely to have consequences for spine formation and synaptic transmission in a neuron-specific way, which would facilitate a fine-tuning of VLDLR regulation in Area X MSNs. This is consistent with data in mice and songbirds (Schulz et al., 2010; DiBattista et al., 2015; Adam et al., 2016). In addition, given that the cellular levels of FoxP2 vary notably with age, song stereotypy, and singing (Haesler et al., 2004; Teramitsu and White, 2006; Miller et al., 2008; Thompson et al., 2013) but FoxP1 and FoxP4 do not (Mendoza et al., 2015) it would be interesting to investigate the consequences of dimerization with FoxP1 and FoxP4 on target gene regulation in the situations when FoxP2 levels vary. In Bengalese finches, *FoxP2* mRNA levels as detected by *in situ* hybridization are also down regulated by singing, but down regulation was not observed for FoxP1 levels (Chen et al., 2013).

The present data on the regulation of *CNTNAP2* by FoxP1/2/4 are interesting in light of the differential activation and repression of target genes by FoxP2 (Vernes et al., 2007). In human SH-SY5Y cells FOXP2 represses *CNTNAP2* (Vernes et al., 2008). In our experiments, HeK293 cells transfected with zebra finch FoxP2 also repressed the *CNTNAP2* promoter activity. In contrast, FoxP1 activated the *CNTNAP2* promoter. FoxP4 did not bind and, as expected, did not regulate the *CNTNAP2* promoter. Of note, when we co-transfected FoxP1/4 or FoxP2/4 there was no significant difference in *CNTNAP2* promoter driven reporter gene activity, suggesting that the presence of FoxP4 prevents regulation by both FoxP1 and FoxP2, even though when present alone, they activate or repress, respectively. This type of differential transcriptional regulation by FOXP proteins has been described for a number of different target genes in Hek293 cells (Sin et al., 2015). In the case where the two components of a dimer have opposing functions when expressed alone, the most parsimonious explanation is that the activation (FoxP1) and the repression (FoxP2) cancel each other out in the reporter assay, but other scenarios are of course possible.

In the brain, CNTNAP2 is also co-expressed in MSN of Area X in zebra finches (Panaitof et al., 2010; Condro and White, 2014). The mRNA amounts of *FoxP2* and *CNTNAP2* in this song nucleus of juvenile male finches and in singing adults are positively correlated. Interestingly, in a zebra finch cell line (Itoh and Arnold, 2011) zebra finch FoxP2 activated the *CNTNAP2* promoter (Adam et al., in review), which is in the opposite direction of the present findings with HeK293 cells. This highlights the plasticity with which FoxP proteins can regulate target genes in different cellular contexts, depending on different binding of co-factors that change the regulation of the same gene (Diamond et al., 1990).

Our data underscore the need to take di- and oligo-merization of the different FoxP proteins more into consideration when trying to understand how mutations of *FOXP1* (Hamdan et al., 2010) and *FOXP2* (Lai et al., 2001) cause disease. Sin et al. (2015) and the present data show that different FoxP proteins can share the same target gene, but that different combinations of proteins can result in opposite effects. It is therefore easily imaginable that a mutation in FoxP1 or FoxP2 could have different effects on the target genes in different neuron types, depending on which other FoxP proteins are co-expressed in the particular cell type (Haesler et al., 2004; Teramitsu et al., 2004; Bowers et al., 2014; Mendoza et al., 2015). This idea is consistent with data from cell lines showing that the subcellular localization deficits caused by FOXP2 mutations can be rescued by co-expression with the wild-type protein (Mizutani et al., 2007; Vernes et al., 2009). It has not been tested whether co-expression with FOXP1 or FOXP4 would have the same effect. If so, it could explain why some tissues might be much more vulnerable to the effects of mutations than others.

Our data do not address which type of protein-protein interaction the homo-and heterodimerization makes use of. FoxP proteins have (at least) two ways to associate or interact: the leucine zipper motif and domain swapping, e.g., the exchange of identical structural elements involving the Forkhead domain (Stroud et al., 2006; Bandukwala et al., 2011; Chu et al., 2011). FoxP proteins with mutations in the leucine zipper protein domain cannot associate (Wang et al., 2003; Li et al., 2004a; Chae et al., 2006), nor bind to DNA or regulate target genes (Li et al., 2004a). For FOXP2 and FOXP3 the inability to dimerize via this domain has also been linked to disorders (Bennett et al., 2001; Li et al., 2007; Roll et al., 2010). Domain swapping has been described for FOXP1/2/3 (Stroud et al., 2006; Bandukwala et al., 2011; Chu et al., 2011) but not so far for FOXP4. It is not known how dimers or multimers of FoxP proteins interact with the regulatory regions of the target genes in any species. In addition, the relative functional importance of associations via the leucine zipper or via domain swapping has not been resolved. One important difference between both types of interaction is that domain swapping has only been reported for FoxP proteins of the same type and not for hetero-associations. In principle, these should also be possible, because all FOXPs share the proline that was reported to be important for domain swapping (Medina et al., 2016) and that other Fox proteins lack. They have an alanine amino acid instead at that position (Stroud et al., 2006; Perumal et al., 2015). In mice the Foxp1/2/4 proteins need to homo- and hetero-dimerize in order to bind DNA and regulate the promoter of the murine CC10 gene, relevant for lung development (Li et al., 2004a). The leucine zipper is a characteristic feature of the FoxP subfamily and is present also in all zebra finch FoxP proteins and episodic positive selection of this domain occurred in some bird species (Mozzi et al., 2016).

Finally, our data are the first to address neural targets regulated by FoxP1 and FoxP4. We tested the binding of two known targets of FoxP2 in zebra finches, *VLDLR* and *CNTNAP2*. All zebra finch FoxP proteins studied bound to the *VLDLR* oligonucleotide, which was previously shown to bind to FoxP2 (Adam et al., 2016). The *VLDLR* oligonucleotide contains a partial FOXP core sequence, **ATTT** (Stroud et al., 2006) and a sequence that resembles the FOXP1 consensus sequence, TT**ATTT**AT (Wang et al., 2003). FOXP2 has similar binding sites (Vernes et al., 2008; Enard et al., 2009; Nelson et al., 2013). All FoxPs share the FOX binding site T**RTTT**AY (Pierrou et al., 1994). The *CNTNAP2* oligonucleotide contains three putative FoxP2 binding sites. One TATTTAT (Enard et al., 2009), and two other sites that have the core ATTT mentioned above Stroud et al. (2006). It is not clear why FoxP4 of zebra finches is not binding, since it would be expected to bind in the presences of the full FoxP consensus (TATTTAT) binding site. However, the binding of FoxP4 is less studied and target genes are not known. To further validate that these putative binding sites are neurally and biologically relevant ChIP data are needed.

In summary, we show that zebra finch FoxP proteins can interact with each other in all combinations in the songbird brain. We show that all neurally expressed FoxPs have the capacity to bind to and regulate the target genes *VLDLR* and *CNTNAP2*. Importantly, different FoxP combinations resulted in specific, differential transcriptional regulation. Together, our data demonstrate how versatile and variable FoxP regulation can be in the neural context.

## Author Contributions

EM and CS: experiment design, analysis of data and wrote the manuscript. EM did all experiments.

## Conflict of Interest Statement

The authors declare that the research was conducted in the absence of any commercial or financial relationships that could be construed as a potential conflict of interest.

## Footnotes

^2^http://www.ebi.ac.uk/Tools/seqstats/emboss_cpgplot/

^3^http://www.bioinformatics.org/sms2/cpg_islands.html
